# A Randomized Controlled Trial of the Korean Version of the Program for the Education and Enrichment of Relational Skills for Young Adults (PEERS®-YA-K) With Autism Spectrum Disorder: A Pilot Study

**DOI:** 10.3389/fpsyt.2021.730448

**Published:** 2021-10-06

**Authors:** Miae Oh, Elizabeth Laugeson, Joo-Hyun Kim, Kyungah Lee, Jeeyun Kim, SeungHa Lee, Bora Lim, Seyoung Cha, Guiyoung Bong, Nan-He Yoon, Geon Ho Bahn, Hee Jeong Yoo

**Affiliations:** ^1^Department of Psychiatry, Kyung Hee University Hospital, Seoul, South Korea; ^2^Semel Institute for Neuroscience and Human Behavior, University of California, Los Angeles, Los Angeles, CA, United States; ^3^Department of Psychiatry, Seoul National University Bundang Hospital, Seongnam, South Korea; ^4^Department of Special Education, Dankook University, Yongin, South Korea; ^5^Maeumddrak Clinical Psychology Center, Seoul, South Korea; ^6^Institute of Education, University College London, London, United Kingdom; ^7^Smile Together Foundation, Seongnam, South Korea; ^8^Division of Social Welfare and Health Administration, Wonkwang University, Iksan, South Korea; ^9^Department of Psychiatry, Seoul National University College of Medicine, Seoul, South Korea

**Keywords:** ASD, cultural adaptations, PEERS®, PEERS®-YA, social skills training

## Abstract

Evidence-based social skills interventions for young adults are limited, despite social difficulties in autism spectrum disorder (ASD) persisting after transition to adulthood. The Program for the Education and Enrichment of Relational Skills for Young Adults (PEERS®-YA) is an evidence-based intervention found to be effective in improving relational skills in young adults with ASD. To translate the original American version of the PEERS®-YA treatment manual into Korean, intensive interviews were performed. Based on results from interviews, several rules of dating etiquette and social activities were modified to be culturally sensitive and linguistically appropriate. Next, young adults diagnosed with ASD (18–35 years of age; IQ > 70) and their social coaches were recruited for the randomized controlled trial (RCT). Participants were randomly assigned either to a treatment group (TG; *n* = 19) or a delayed treatment group (DTG; *n* = 18). In the analysis of group differences in the TG and DTG, social skills knowledge was improved. The within group analyses showed positive effects of improving social skills knowledge on reducing depression and anxiety symptoms. After modest cultural adaptations focusing on dating and social activities, the implementation of the PEERS®-YA-K was found feasible for the Korean community. This is one of only a few cross-cultural validation trials establishing evidence-based treatment in young adults with ASD.

**Clinical Trial Registration:** This trial was registered at ClinicalTrials.gov, identifier: NCT03310775.

## Introduction

Autism or Autism Spectrum Disorder (ASD) is a neurodevelopmental disorder characterized by communication impairments, social skills deficit, and the presence of restricted and repetitive behaviors and impaired imagination (1). ASD is a lifelong condition that persists after transition to adulthood and often requires additional support for establishing an independent life. Over 40 years ago, in a landmark study by (2), 96 adults with ASD in their 20 and 30s were observed. The study revealed that the majority were living with their parents or in sheltered communities and were highly dependent on others; only 11 individuals had jobs; one was in college; and only one was married. More recent studies have found that young adults with ASD experienced social isolation, unemployment, financial distress, and a high degree of dependency on others (3–6). Many argue that deficits in social skills are often the cause of these pervasive deficits (7).

Deficits in relational skills are one of the most challenging areas for young adults with ASD (8). Many researchers have reported that young adults with ASD desire to have relationships but lack the appropriate skills and knowledge to initiate them (9, 10). Although many adults with ASD long for a romantic partner when they experience sexual fantasies and desires, it is difficult for them to initiate and maintain romantic relationships (11). The complexity involved in forming intimate and romantic relationships is further compounded by delay in the development of social functioning in individuals with ASD (12). Even for typically developing adults, many would argue that understanding and utilizing successful strategies to initiate and maintain romantic relationships are challenging. Behaviors such as choosing an appropriate partner, discriminating between appropriate and inappropriate romantic behaviors and gestures, and interpreting and responding appropriately to a partner's response are crucial elements to successful relationships (13). Adults on the autism spectrum often respond inappropriately, which may be mistaken for aggressive sexual behavior, such as stalking, unwanted contact, exhibition, fetishism, and sexual compulsion, regardless of their intentions (12).

Social skills training as a group intervention has been shown to be an effective method of treatment for individuals with ASD, especially those with average and above average intellectual functioning (14). However, evidence-based social skills interventions for young adults are limited compared to those for children and adolescents (8). Cognitive enhancement therapy (CET) is also an evidence-based intervention for individuals with ASD. Eack et al. conducted a single-blind trial with 54 adults with ASD and compared CET to active enriched supportive therapy and found that CET significantly improved cognitive symptoms and employment (15). The Program for the Education and Enrichment of Relational Skills for Young Adults (PEERS^®^-YA) is other evidence-based interventions known to be effective in improving relational skills for young adults with ASD (13). The PEERS^®^-YA focusses on skills related to making friends, developing romantic relationships, and managing conflict and rejection. The program has 16 sessions and is designed for high functioning adults with ASD. The PEERS^®^-YA provides the same intervention guidance to ASD adults as it does to their social coaches, such as parents, adult siblings, other family members, friends, partners, and peer mentors, to provide support through continuous practice and reinforcement. Treatment evidence of the effectiveness of the original version of PEERS^®^-YA has been established through two randomized controlled trials (RCTs) using delayed treatment groups (16, 17). The young adults who participated in the PEERS^®^-YA showed significant improvements in overall social skills, which included social skills knowledge, social motivation, social communication, assertiveness, and responsibility. Improvements were also seen in decreasing repetitive behaviors, such as perseverating on topics of interest and restricted interests. The effect was re-examined and replicated in larger samples in the United States and showed improvement in social anxiety as well as social relational skills (18).

It is assumed that the contents of social skills training programs might have to be modified since research suggests that the social skills required to create and maintain social relationships may differ based on culture (19). In a previous RCT of the Korean version of the PEERS^®^ social skills intervention for teenagers with ASD (20), the research team surveyed 447 adolescents within the Korean community to modify the curriculum to be culturally valid. In the survey, participants reported that the most popular examples of social networking sites, common extracurricular activities, and classification of peer groups and crowds in Korea were different from those of American teenagers. This feedback was used to culturally adapt the curriculum to be more relevant to the Korean youth culture. An RCT was subsequently performed with a modestly modified curriculum for 47 teenagers aged 12–18 years who were randomly assigned to a treatment group or a delayed treatment control group. Within 14 weeks of treatment, participants showed a significant improvement in social skills knowledge *via* the total score of the Test of the Adolescent Social Skills Knowledge-Revised, improved interpersonal skills domain scores *via* the Korean version of the Vineland Adaptive Behavior Scale, a decrease in depressive symptoms on the Child Depression Inventory, and a decrease in ASD symptoms on the Autism Diagnostic Observation Schedule (ADOS), which were comparable to the original findings for PEERS^®^ for adolescents in North America. This study was one of the few cross-cultural validation trials to support the use of an evidence-based treatment for adolescents with ASD outside of North America.

Given the process of cultural adaptation in our previous research, we naturally assumed that the social conventions for young adults described in the American version of the PEERS^®^ for Young Adults manual might also need cultural modification based on the lives of young adults in the Korean community. In particular, cultural modifications focused on dating etiquette, which are unique to the young adult curriculum, would be necessary since there might be cultural differences in romantic relationships between Korean young adults and those from Western cultures such as in the United States. This notion is supported by (21), who reported that Western cultures have a stronger desire for romantic relationships and rely more heavily on these relationships than non-Western cultures possibly because non-Western cultures, such as in Korea, have other strong sources of intimacy, such as from parents and siblings. In Asian cultures, most parents raise their children and then expect to be cared for by them when they reach old age (22). As an example, according to the National Survey on Fertility and Family Health and Welfare in Korea (23), among unmarried people aged 20–44, 72.7 % of men and 78.8% of women were living with their parents. To further highlight this point, Seepersad et al. compared the romantic relationships of 227 young adults from America and Korea who ranged in age from 18 to 25 (24). The study revealed that American young adults reported higher levels of romantic loneliness when not in a romantic relationship and a greater degree of closeness in romantic relationships than Korean young adults. In a separate study comparing Greek and American cultures also showed that culture impacts social desirability and shapes partnering norms and romantic behaviors (25). Thus, the social etiquette associated with developing and maintaining romantic relationships appears to be culturally sensitive and requires further investigation in the cross-cultural validation of social skills intervention.

The objective of this study was to examine the effectiveness of the validation of the Korean version of the PEERS^®^-YA (PEERS^®^-YA-K) using an RCT design to improve the social skills of young adults with ASD. We applied the same waitlist control design using a delayed treatment group as in the original study by Laugeson (13), which was the first replication of PEERS^®^-YA outside the US. Acknowledging the trans-cultural differences in social norms in young adults, we used the translated version of PEERS^®^-YA, through cross-cultural modification for culturally sensitive contents. It was hypothesized that the Korean version of the PEERS^®^-YA-K would improve relational skills in young adults with ASD, even after modification for cultural differences. We anticipated that there would be an improvement with the PEERS^®^-YA-K in social skills, social skills knowledge, social responsiveness, and a decrease in comorbid psychosocial distress for young adults with ASD.

## Methods

### Process 1–Translation and Adaptation of the Peers^®^-YA Treatment Manual

#### Translation

The American version of the PEERS^®^-YA Treatment Manual^13^ was translated into Korean by two PEERS^®^ Certified Providers. The translated manual was meticulously reviewed by six autism specialists, including child psychiatrists and clinical psychologists. The structure and content of the English version were maintained; however, content that was found to be culturally sensitive in the Korean adaptation of the PEERS^®^ for Adolescents curriculum was modified in the relevant portions of the adult curriculum.

#### Adaptation

An intensive interview was performed to further adapt the intervention and incorporate ecologically valid social skills specific to typical adults in Korea. A total of 29 typically developing young adults (11 males, a mean age of 25 years; range 19–34; *SD* = 3.70) were recruited for the interviews. Thirty-three questions focusing on the dating etiquette of young adults along with 14 questions concerning social groups/activities and strategies for handling bullying were discussed. After the interview, the research team categorized and determined the items which had to be changed, edited therapists' scripts, and added appropriate examples of role play materials. The detailed adaptation process, modifications, and a summary of the changes to the Korean version of PEERS^®^-YA Treatment Manual are described in Supplementary Material 1 and Supplementary Table 1.

#### Training and Staff Roles

The PEERS^®^-YA required two group leaders, one for the social coaching group and the other for the young adult group. The social-coach group leaders included a board-certified child psychiatrist who is a PEERS^®^-YA Certified Provider and a special educator with specialty training in the treatment of ASD and group therapy. The young-adult group leaders included licensed clinical psychologists, and one of them was certified for conducting PEERS^®^ for adolescents. After 20 h of training in the treatment team, treatment leaders observed each session of the treatment conducted in Korean by the PEERS^®^-YA Certified Provider, and subsequently established reliability with the treatment protocol prior to actual treatments with the participants. Two or three behavioral coaches in each young-adult group conducted role-play demonstrations of targeted skills, provided social coaching with performance feedback during behavioral rehearsal exercises, and monitored treatment fidelity and homework compliance throughout the sessions.

#### Intervention Content and Structure

The PEERS^®^-YA consisted of 16 weekly 90-min sessions. Social coaches and the young adults were given concurrent sessions in separate rooms and each session was led by five trained treatment leaders. The didactic lessons focused on the social skills necessary for developing and maintaining friendships, romantic relationships, and managing peer conflict and rejection. The lessons included conversational skills, electronic forms of communication, developing friendship networks and finding sources of friends, appropriate use of humor, peer entry and exiting strategies, organizing and having successful get-togethers with friends, handling teasing and chronic bullying in the school or workplace, managing peer pressure, conflict resolution, strategies related to dating etiquette, showing romantic interest, asking someone on a date, going on dates, and general dating guidelines. The detailed processes and characteristics of the PEERS^®^-YA intervention are described by Laugeson (13).

### Process 2–Evaluation of the Effects of the Korean Version of Peers^®^-YA

#### Recruitment of Participants

The participants were recruited from the Child and Adolescent Psychiatric Clinic at Seoul National University Bundang Hospital, through advertisements at the Smile Together Foundation (a local advocacy institute of ASD), a community of child and adolescent psychiatrists, an internet group of families of youth with ASD, and a website for Seongnam Child and Adolescent Psychiatry Mental Health Care Center. Inclusion criteria for young adults included: (1) age between 18 and 35 years, (2) high school graduation or expected to graduate by the time the intervention starts, (3) experiencing social difficulties as recognized by young adults and/or social coaches, (4) previously diagnosed with or possibly having a diagnosis of ASD, (5) verbally fluent, (6) full scale intelligence quotient (FSIQ) ≥ 70 by the Korean Wechsler Adult Intelligence Scale-IV [K-WAIS-IV; (26)], and (7) substantially motivated to participate in the intervention program. Exclusion criteria included: (1) history of major mental illness (e.g., schizophrenia, bipolar disorder, psychosis, and risk of self-harm), (2) significant physical disabilities including visual and/or hearing impairments, (3) clinically significant neurological or physical disease that would preclude participation in group-based social activities, and (4) difficulties for social coaches to understand and cooperate with the intervention.

The study was approved by IRB of Seoul National University Bundang Hospital (IRB no. B-1611/371-303) and the study was registered at ClinicalTrials.gov (identifier: NCT03310775). Written informed consent was provided by all young adults and social coaches who participated in the present study.

#### Screening and Randomization Process

Participants were screened for eligibility by telephone interview using the Phone Screening Interview in the PEERS^®^-YA treatment manual. Motivation, social problems related to friendships, willingness to follow the rules in the group, comorbidities, and inclusion and exclusion criteria were discussed during the telephone interview.

**Figure 1:**
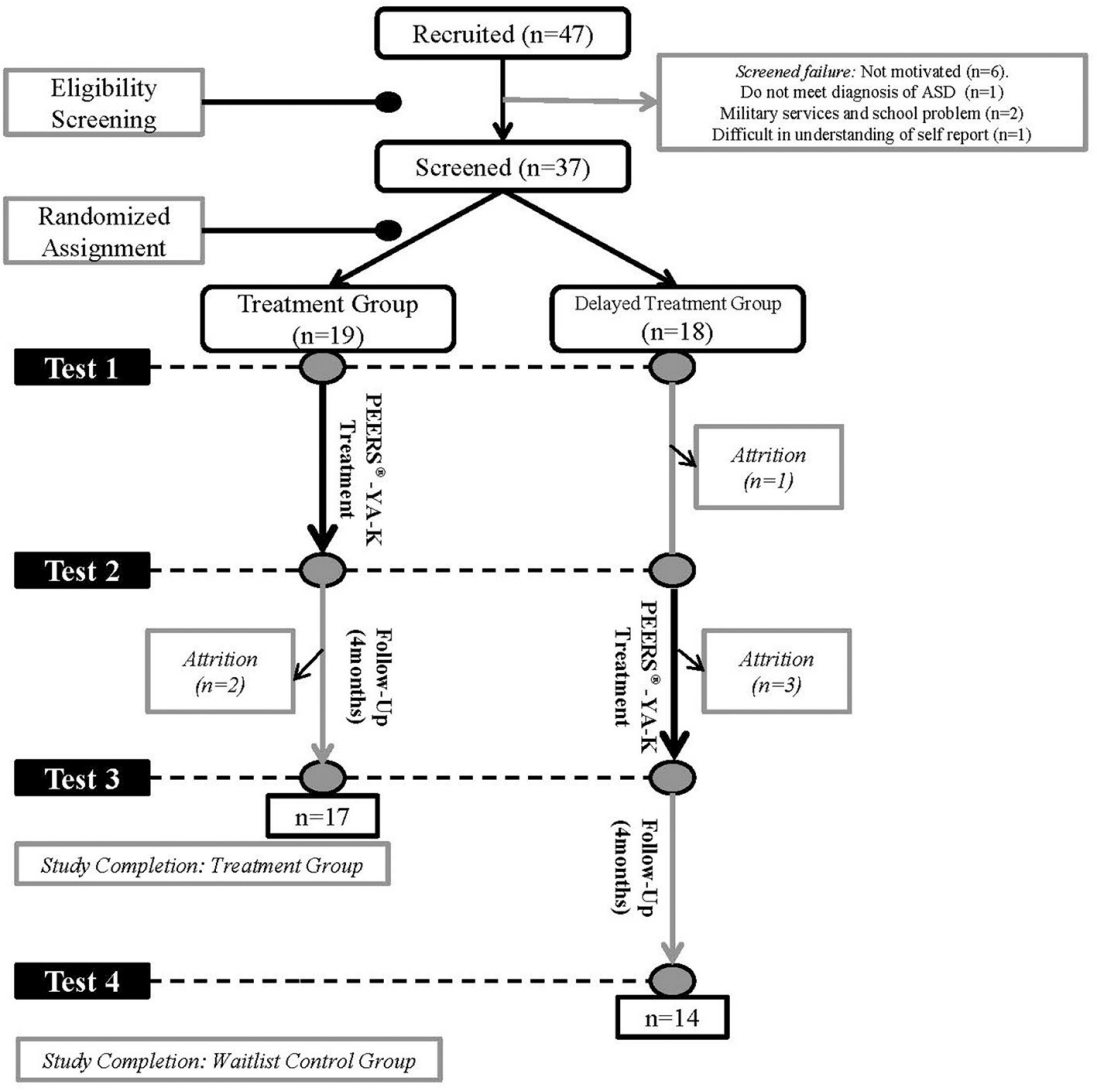
Subject recruitment, assignment, and assessment procedures.

Diagnosis of ASD was confirmed for screened individuals by two board-certified child psychiatrists based on diagnostic criteria of the fifth edition of the Diagnostic and Statistical Manual of Mental Disorders (1). The Autism Diagnostic Observation Schedule, second edition [ADOS-2; (27)] and the Autism Diagnostic Interview-Revised [ADI-R; (28)] were used for supporting diagnostic procedures. Participants meeting eligibility criteria were randomly assigned to a treatment group (TG) or a delayed treatment group (DTG) using randomized block design. We divided each group into two separate treatment groups of ~9–10 young adults. Formal social skills training, social skills group therapy, and starting new therapy were prohibited for the DTG during the waiting period but individual counseling, general follow-up visits, and pharmacotherapy were allowed. The study design is shown in Figure 1.

#### Participants

A total of 48 young adults ranging from 18 to 35 years old and their social coaches were recruited for the RCT. Two participants were excluded during the randomization procedure: one did not meet the diagnosis of ASD and one had difficulty understanding the self-report scales. Eight participants withdrew the application for participation: six were not adequately motivated, one had a problem with school, and one had to fulfill a military commitment. A total of 38 participants were screened, but one participant refused to be randomized to the DTG. As a result, 37 participants were randomized: 19 were assigned to TG and 18 to DTG. 31 participants (TG = 17, DTG = 14) completed the 16-week trial. In the TG, two participants dropped out during the 4 month follow-up period because they either joined the army or refused to complete the test. In the DTG, one participant dropped out before the intervention began due to lack of motivation and three dropped out in the middle of the intervention due to aggravation of depressive symptoms and a suicide attempt, lack of motivation, and lack of availability due to work commitments.

Only one participant was female, and among the total participants, one had graduated from middle school; 21 had graduated from high school; and five had graduated from college. Twenty-four participants were college students; 11 were unemployed; one was in military service; and one had a job. Twenty-three social coaches were mothers (85%), four were fathers (15%), and one was a mentor of the young adult. The attendance rate was 96.5% and the overall homework completion rate was 48.5%.

#### Treatment Fidelity

Treatment fidelity was monitored to ensure that all processes of the intervention were completed. At each session, treatment team members and volunteers comprising undergraduate students in psychology and special education checked intervention components to assess that the treatment was implemented as planned. The score of the treatment fidelity was calculated by dividing the number of delivered intervention items by the total number of planned intervention items. The average score of the treatment fidelity in each group was 0.95.

### Measures

Every participant and their social coach in the TG completed outcome measures prior to the start (test 1) and after the completion of the intervention (test 2). Participants and social coaches in the DTG completed outcome measures upon entering the study (test 1), prior to the start of treatment (test 2), and after the completion of the treatment (test 3). Four months after completing the program, both groups completed outcome measures to evaluate the maintenance of the treatment effects (TG: test 3; DTG: test 4). Assessment schedules for both groups are shown in Figure 1. Primary outcome measures were defined as assessments used to quantify improvements by PEERS^®^-YA-K in problems directly related to ASD. Secondary outcome measures were assessed depressive symptoms and adaptive functioning. Lastly, other variables that we assumed would potentially improve after treatment and influence the primary or secondary outcomes were defined as exploratory measures.

#### Primary Measures

##### Autism Diagnostic Observation Schedule, Second Edition [ADOS-2 Module 4; (27)]

The ADOS-2 is a standardized test designed to diagnose ASD by observing their communication and social behaviors during semi-structured play and/or an interview. The ADOS-2 is composed of four modules. Each module is selected according to the expressed language level and chronological age of participants and requires 35–40 min to administer. The current study used ADOS-2 module 4. Module 4 of the ADOS-2 is used to diagnose verbally fluent adolescents and adults. It is scored on five domains: language and communication, reciprocal social interaction, imagination, stereotyped behaviors, restricted interests, and other abnormal behaviors. In this study, raters on the ADOS-2 were blinded to participants' groups and the timing of testing. Internal consistency, as examined using Cronbach's alpha, was 0.752.

##### Test of Young Adult Social Skills Knowledge [TYASSK; (13)]

The TYASSK is used to evaluate the change in young adults' knowledge of specific social skills taught during the intervention period with two items derived from each of the lessons. Scores range from 0 to 30, with higher scores reflecting more knowledge of social skills. The English version of the TYASSK was translated into Korean with the author's permission, and then back-translated into English by a bilingual translator unrelated to this study. The translation was then reviewed again by the Korean PEERS^®^ research team. The α for TYASSK was 0.403.

##### Korean Version of the Social Skills Rating System [K-SSRS; (29, 30)]

The SSRS is a multi-rater feedback questionnaire to assess social skills, problematic behaviors, and academic competence. The SSRS for secondary students, originally developed for adolescents. It was modified for college students (K-SSRS: college level) and its reliability and utility were verified by Moon. The K-SSRS consists of 27 items which are rated on a Likert scale from 1 (“not at all”) to 7 (“almost always”). Cronbach's alpha of self-report and parent-report were 0.910 and 0.920, respectively.

##### Social Responsiveness Scale-2 [SRS-2; (31)]

The SRS-2 is used to screen children at risk of ASD and consists of measures regarding the child's social interactions, communication, and stereotyped behaviors. Individual items are rated on a Likert scale from 1 (“not at all”) to 4 (“almost always”) to measure the severity of ASD symptoms as they occur in natural social settings. The individual is classified as being at a high risk of ASD when the score is >75. In this study, we used the adult form reported by the caregiver and the self-report form. Cronbach's alpha of self-report and parent-report were 0.946 and 0.921, respectively.

#### Secondary Measure

##### Korean Version of the Vineland Adaptive Behavior Scale, Second Edition [K-VABS; (32, 33)]

The K-VABS is an interview survey and measures adaptive behaviors of individuals such as personal and social skills used for everyday living. The K-VABS contains 5 domains and the five domains are, communication, daily living skills, socialization, motor skills, and maladaptive behavior. Behaviors are rated on a 0 (a skill that is not used by the individual) to 2 (a skill used most of the time) rating scale. The current study used only the communication, daily living skills, and socialization domains. The α of the K-VABS was 0.812.

##### Beck Depression Inventory [BDI; (34, 35)]

The BDI is a 21-question, rated from 0 to 3 in terms of intensity, self-report inventory. The items are used to assess the intensity of 21 depression-related symptoms and attitudes. The maximum score of the BDI is 63 points, with a suggested interpretation of, minimal range = 0–13, mild depression = 14–19, moderate depression = 20–28, and severe depression = 29–63. The Korean version of the BDI was developed by Han et al. α of the BDI was 0.942.

#### Exploratory Measures

##### Quality of Socialization Questionnaire [QSQ; (13)]

The QSQ is a self- and caregiver-report measure that assesses the frequency of hosted get-togethers, hosted dates, invited get-togethers, and invited dates over the previous month. The QSQ was translated into Korean and back-translated into English with the author's permission. The alpha value of QSQ was calculated by coding 0 for each item with “0 times” and 1 for each item with “more than once” to be converted as a binary variable. Cronbach's alpha of self-report and parent-report were 0.781 and 0.643, respectively.

##### Social Communication Questionnaire [SCQ; (36)]

The SCQ is a primary caregiver/parent reported measure assessing symptomatic ASD behaviors. The SCQ consists of two forms: “Current Form” and “Lifetime Form.” They are both composed of 40 “yes or no” items. Each item is scored 1 or 0, except the first item which does not receive a score and determines whether a patient is verbal or non-verbal. The total score range is 0 to 39 for verbal people and 0 to 33 for non-verbal people. The current study used the “Life Form” of the SCQ to evaluate baseline differences and the “Current Form” to evaluate treatment effectiveness. α for the SCQ was 0.791.

##### Beck Anxiety Inventory [BAI; (37)]

The BAI measures common symptoms of anxiety such as numbness and tingling, sweating unrelated to heat, and fear of the worst happening. The BAI is a 21-question self-report inventory with questions rated from 0 to 3. The maximum score of the BAI is 63 points, with 0–7 indicating minimal anxiety, 8–15 indicating mild anxiety, 16–25 implying moderate anxiety, and 26–63 indicating severe anxiety. The BAI shows good internal consistency with an α of 0.956.

##### State and Trait Anxiety Inventory [STAI-T and STAI-S; (38)]

The State-Trait Anxiety Inventory (STAI-Y) has 40 items, 20 items allocated to each of the STAI -S and STAI -T subscales. The STAI-S asks respondents to indicate how they feel about anxiety, tension, and nervousness at the present time. The STAI-T asks the respondents to indicate how anxious they feel in daily life. The STAI-S score shows the strength of an individual's anxiety response at the present time and the STAI-T score shows the frequency of a specific anxiety response. The score range for each subscale is 20–80. A higher score indicates greater anxiety. Scores of 52–56 indicate mild, 57–61 moderate, and 62 and over indicate severe anxiety for the STAI-S. Scores of 54–58 indicate mild, 59–63 moderate, and 64 and over indicate severe anxiety for the STAI-T. The α for the STAI-S and STAI-T were 0.836 and 0.853, respectively.

### Statistical Analyses

Baseline characteristics were compared between the TG and DTG with independent samples *t*-tests for continuous variables and Fisher's Exact Test for categorical variables. Changes in outcomes between baseline and post-intervention and the maintenance of treatment effects in both groups were examined with paired *t*-tests. Post-intervention follow-up measurements were analyzed using repeated measures analysis of variance (ANOVA) comparing both between-group and between-time differences in outcomes by a measured time point interaction. *Post-hoc* comparisons were applied with the Bonferroni correction, Fisher's LSD test, Scheffe's Test, and Tukey's Studentized Range Test to correct for multiple comparisons in the ADOS-2 raw subtotals and CSS scores. The significance of difference was interpreted with treatment x time interaction from the analysis, and the treatment effect size was assessed with Cohen's d for the interaction (39). All statistical analyses were conducted using SAS version 9.4 (SAS Institute, Cary, NC, USA) and statistical significance was defined as *p* < 0.05.

## Results

### Participant Characteristics

Of the 37 participants, 31 completed the study with a completion rate of 83.78%. Table 1 shows the summary of the baseline characteristics and mean demographics for both groups. The average age of the TG was 23.3 years (*SD* = 3.7) and DTG was 23.5 years (*SD* = 4.1). The mean FSIQ of TG and DTG were 100 and 99.1, respectively (*SD* = 14.6, 16.2); Verbal Comprehension Index = 109.5 (*SD* = 15), 105.9 (*SD* = 17); Working memory index (WMI) = 98.5 (*SD* = 19.3), 98.9 (*SD* = 15.3); Processing speed index (PSI) = 83.3 (*SD* = 15.8), 81.8 (*SD* = 17.5), respectively. There were no significant differences in demographic characteristics between the two groups. The social communication skills directly observed by module 4 of the ADOS-2 did not show significant differences between the two groups. Social skills knowledge, social skills, social functioning, and social responsiveness of the participants evaluated by the TYASSK, SCQ, K-SSRS, QSQ, and K-VABS (respectively) were not statistically different between the TG and DTG; however, the SRS-2 rated by young adults and the frequency of hosted dates measured by the QSQ reported by parents showed differences between the groups (*p*'s <0.05). The effect of the SRS-2 and QSQ was considered in the analysis of results. Depressive and anxiety symptoms evaluated by the BAI, BDI, STAI-S, and STAI-T, and SCL-90 showed no significant statistical differences between the TG and DTG participants.

**Table 1:** Mean demographic and baseline variables.

		**TG**	**(*n* = 19)**	**CG**	**(*n* = 18)**	***T***	**df**.	***p*-value**
		**Mean, *n***	**SD, %**	**Mean, n**	**SD, %**			
Age		23.3	3.7	23.5	4.1	0.18	35	0.855
Intelligence	FSIQ (Standardized score)	100.0	14.6	99.1	16.2	−0.19	35	0.853
	VCI (Standardized score)	109.5	15	105.9	17	−0.62	30	0.542
Education (Fisher's Exact Test)	Middle school	0	0.0	1	5.6	–	–	0.490
	High school	17	89.5	14	77.8			
	College	2	10.5	3	16.7			
Medication (Fisher's Exact Test)		12	63.2	5	27.8	–	–	0.080
ADOS 2 (module 4)	Communication	3.6	1.2	3.9	1.3	0.87	35	0.392
	Social interaction	8.5	2	9.3	2.1	1.13	35	0.265
	Stereotyped behaviors and restricted interests	1.0	0.0	1.1	0.3	1.46	17	0.163
	Imagination	0.8	1.3	0.4	0.5	−1.06	24	0.299
ADOS 2 (calibrated severity scores)	Social affect	11.8	2.7	13.6	3.1	1.78	35	0.083
	RRB	2.4	1.7	2.0	1.1	−0.84	33	0.409
	Total	14.3	3.5	15.5	3.6	1.06	35	0.295
	Comparison Score	7.6	1.7	8.2	1.4	1.16	35	0.253
Social skills measures	TYASSK	16.6	3.2	17.5	3.4	0.84	35	0.404
	SCQ, lifetime	21.2	7.5	16.8	8.9	−1.60	33	0.119
	SSRS by young adults	112.4	25.2	105.6	22.2	−0.88	35	0.386
	SRS-2 by young adults	144.4	18.5	159.7	21.6	2.32	35	0.026^*^
	SSRS by parents	86.9	24.8	90.5	0.670	0.43	34	0.670
	SRS-2 by parents	150.4	21.2	155.9	26.8	0.70	35	0.491
QSQ by young adults (Fisher's Exact Test)	Hosted get together (≠0)	11	57.9	9	50.0			0.746
	Hosted date (≠0)	3	15.8	0	0.0			0.230
	Invited get together (≠0)	7	36.8	6	33.3			1.000
	Invited date (≠0)	3	15.8	0	0.0			0.230
QSQ by parents (Fisher's Exact Test)	Hosted get together (≠0)	12	63.2	10	55.6			0.743
	Hosted date (≠0)	5	26.3	1	5.6			0.180
	Invited get together (≠0)	7	36.8	6	33.3			1.000
	Invited date (≠0)	2	10.5	1	5.6			1.000
K-VABS	Communication	70.3	13.7	68.5	17.5	−0.34	33	0.735
	Daily living skills	70.9	19.1	82.5	17.9	1.85	33	0.073
	Socialization	62.6	12.3	70.2	15.1	1.64	33	0.111
Depressive and anxiety measures	STAI-S	54.8	10.7	54.2	8.5	−0.19	34	0.852
	STAI-T	57.7	9.9	54.2	9.0	−1.13	34	0.267
	BAI	32.4	12.4	30.2	11.4	−0.11	34	0.917
	BDI	15.9	10.6	14.3	12.9	−0.40	33	0.692

* *p <0.05*.

*ADOS, Autism Diagnostic Observation Schedule; BAI, Beck Anxiety Inventory; BDI, Beck Depression Inventory; FSIQ, Full Scale Intelligence Quotient; K-VABS, Korean version of the Vineland Adaptive Behavior Scale; RRB, Restricted and Repetitive Behaviors; SCQ, Social Communication Questionnaire; SRS-2, Social Responsiveness Scale-2; SSRS, Social Skills Rating System; STAI-S and STAI-T, State and Trait Anxiety Inventory; TYASSK, Test of Young Adult Social Skills Knowledge; QSQ, Quality of Socialization Questionnaire; VCI, Verbal Comprehension Index*.

#### Comparisons Between TG and DTG

To evaluate the effectiveness of treatment more accurately in each group, repeated measures ANOVAs were performed for differences in variables between the TG and DTG before and after treatment, with a condition (TG vs. DTG) × time (pretest vs. post-test) analysis. Five models were constructed to control covariates that potentially affected treatment outcomes: in model I, there were no covariates; model II controlled for age; model III controlled for the variables included in model II and added FSIQ; model IV controlled for the variables included in model III and added medication; and model V controlled for the variables included in model IV and added homework completion. We observed a significant influence in TYASSK scores resulting from the interaction effect between TG and DTG (*F* = 4.80, df = 26, *p* = 0.038 for Model V). Among covariates controlled in the analytic models, age had a significant effect on ADOS-2 calibrated severity scores in total (*F* = 6.37, df = 25, *p* = 0.018 for Model V), and FSIQ on BDI (*F* = 7.65, df = 23, *p* = 0.011 for Model V) and K-VABS communication (*F* = 4.66, df = 20, *p* = 0.043 for Model V). Medication significantly affected ADOS-2-stereotyped behaviors and restricted interests (*F* = 5.01, df = 25, *p* = 0.034 for Model V), BDI (*F* = 5.79, df = 23, *p* = 0.025 for Model V), and QSQ by parents-invited get together (*F* = 4.45, df = 22, *p* = 0.047 for Model V). Homework completion had significant effect on K-SSRS by young adults (*F* = 6.98, df = 26, *p* = 0.014 for Model V), K-SSRS by parents (*F* = 10.28, df = 26, *p* = 0.004 for Model V), and SCQ current (*F* = 7.57, df = 22, *p* = 0.011 for Model V). Mean differences and significance for time x treatment differences of outcome variables in the TG and DTG are provided in Table 2.

**Table 2:** Mean differences and effect size with significance for time x treatment differences of each item.

		**Mean difference**		**I**		**II**		**III**		**IV**		**V**		**Cohen's d^†^**
		**(T2–T1)**							
		**TG**	**CG**	**F**	***p***	**F**	***p***	**F**	***p***	**F**	***p***	**F**	***p***	
ADOS 2 (module 4)	Communication	−0.4	−0.3	0.05	0.820	0.10	0.758	0.09	0.761	0.05	0.820	0.02	0.899	0.06
	Social interaction	−0.1	−0.2	0.06	0.802	0.04	0.835	0.04	0.838	1.82	0.188	1.26	0.272	−0.06
	Stereotyped behaviors and restricted interests	0.0	0.0	0.00	1.000	0.00	0.977	0.00	0.978	0.25	0.622	0.10	0.751	0.00
	Imagination	−0.3	−0.1	0.46	0.501	0.51	0.482	0.50	0.483	0.65	0.427	1.44	0.241	0.20
ADOS 2 (calibrated severity scores)	Social affect	−0.2	−1.0	1.22	0.277	1.15	0.290	1.10	0.302	3.71	0.064	2.89	0.101	−0.26
	RRB	−0.4	−0.2	0.45	0.509	0.52	0.477	0.54	0.469	0.67	0.421	2.00	0.169	0.16
	Total	−0.6	−1.3	0.64	0.429	0.51	0.482	0.49	0.489	2.37	0.135	1.23	0.278	−0.19
	Comparison Score	−0.4	−0.8	1.02	0.320	0.99	0.327	0.95	0.338	2.45	0.129	2.13	0.157	−0.24
Social skills measures	TYASSK	5.4	0.8	10.63	0.003^**^	10.27	0.003^**^	9.95	0.004^**^	6.32	0.018^*^	4.80	0.038^*^	1.38
	SCQ, current	−1.9	0.9	1.57	0.219	1.73	0.199	1.70	0.203	0.77	0.388	1.24	0.277	0.55
	SSRS by young adults	6.6	−2.4	3.01	0.092	2.87	0.100	2.78	0.106	1.50	0.231	2.56	0.122	0.38
	SRS-2 by young adults	−3.8	−7.4	0.29	0.596	0.34	0.565	0.33	0.570	0.10	0.750	0.00	0.971	−0.17
	SSRS by parents	2.1	2.9	0.01	0.910	0.01	0.926	0.01	0.923	0.66	0.424	0.34	0.564	−0.03
	SRS-2 by parents	2.9	−4.8	0.99	0.327	0.97	0.332	0.93	0.341	0.06	0.802	0.03	0.864	−0.32
QSQ by young adults	Hosted get together	0.8	−0.2	0.77	0.388	0.82	0.372	0.78	0.383	0.95	0.340	1.34	0.259	0.42
	Hosted date	−0.4	0.5	2.12	0.156	2.45	0.129	2.35	0.137	0.81	0.377	1.30	0.267	−0.98
	Invited get together	0.6	−0.5	1.86	0.185	1.49	0.234	1.64	0.213	0.53	0.477	0.45	0.509	0.68
	Invited date	−0.4	0.0	0.96	0.335	0.90	0.352	0.81	0.377	0.40	0.532	0.71	0.412	−0.42
QSQ by parents	Hosted get together	1.2	−0.1	1.78	0.191	1.91	0.176	1.91	0.177	0.75	0.394	1.25	0.274	0.54
	Hosted date	−0.1	0.7	1.68	0.204	1.75	0.195	1.84	0.184	1.16	0.291	0.63	0.433	−0.72
	Invited get together	0.5	0.0	0.78	0.385	0.73	0.400	0.65	0.426	0.25	0.622	0.42	0.526	0.29
	Invited date	0.1	0.2	0.05	0.830	0.09	0.763	0.08	0.774	0.00	0.978	0.03	0.856	−0.12
K-VABS	Communication	2.4	1.8	0.01	0.919	0.03	0.866	0.05	0.824	0.15	0.706	0.01	0.941	−0.04
	Daily living skills	1.4	−3.3	0.72	0.403	0.96	0.336	0.93	0.343	0.11	0.741	0.64	0.434	−0.25
	Socialization	2.1	0.1	0.23	0.637	0.38	0.541	0.30	0.587	0.41	0.530	0.22	0.648	−0.14
Depressive and anxiety measures	STAI-S	−0.4	−0.3	0.00	0.981	0.01	0.933	0.01	0.919	0.02	0.903	0.02	0.902	0.01
	STAI-T	−2.7	−0.8	0.60	0.445	0.63	0.434	0.68	0.416	0.26	0.611	0.22	0.640	0.20
	BAI	−2.1	1.1	0.84	0.368	0.87	0.358	0.85	0.363	0.64	0.430	0.85	0.366	0.27
	BDI	−3.9	0.8	1.74	0.197	1.69	0.204	1.66	0.209	1.64	0.212	1.86	0.186	0.40

* *p <0.05*,

** *p <0.01*.

† *Effect sizes were calculated for model I*.

*Model I no covariates*.

*Model II controlled for age*.

*Model III controlled for the variables included in model II and added FSIQ*.

*Model IV controlled for the variables included in model III and added medication*.

*Model V controlled for the variables included in model IV and added homework completion*.

*ADOS, Autism Diagnostic Observation Schedule; BAI, Beck Anxiety Inventory; BDI, Beck Depression Inventory; FSIQ, Full Scale Intelligence Quotient; K-VABS, Korean version of the Vineland Adaptive Behavior Scale; RRB, Restricted and Repetitive Behaviors; SCQ, Social Communication Questionnaire; SRS-2, Social Responsiveness Scale-2; SSRS, Social Skills Rating System; STAI-S and STAI-T, State and Trait Anxiety Inventory; TYASSK, Test of Young Adult Social Skills Knowledge; QSQ, Quality of Socialization Questionnaire; VCI, Verbal Comprehension Index*.

#### Pre-treatment and Post-treatment Comparisons: Whole Group Analysis

Paired *t*-tests were conducted to compare changes in outcomes over time. Outcome variables were tested before, after, and 4 months post-treatment. On the sociality outcome measures, a paired *t*-test revealed significant differences in total TYASSK scores and total scores of the K-SSRS by young adults before and after PEERS^®^-YA-K treatment (*p*'s <0.05). The communication domain of the K-VABS also improved after treatment (*p* < 0.05). No statistically significant differences were found for ADOS-2, QSQ, and depressive and anxiety measures. A summary of pretreatment and post-treatment comparisons is presented in Table 3 and the result of the *post-hoc* test of ADOS-2 and ADOS calibrated severity scores is shown in Table 4.

**Table 3:** Comparison of pre-treatment, post-treatment, and 4 months follow-up outcome measures.

		**Pre-treatment** **(*****n*** **=** **36)**		**Post-treatment** **(*****n*** **=** **32)**		**Follow-up** **(*****n*** **=** **31)**		**Pre-post**			**Pre-F/U**		
		**Mean**	**SD**	**Mean**	**SD**	**Mean**	**SD**	***t***	**df**.	***p***	***t***	**df**.	***p***
ADOS 2 (module 4)	Communication	3.6	1.3	3.3	1.0	3.4	1.3	0.95	30	0.351	0.63	29	0.536
	Social interaction	8.8	2.3	8.1	2.3	7.5	2.1	1.22	30	0.231	2.51	29	0.018^*^
	Stereotyped behaviors and restricted interests	1.1	0.2	1.0	0.2	1.0	0.2	1.00	29	0.326	0.00	21	1.000
	Imagination	0.6	1.0	0.4	0.6	0.7	0.8	1.68	30	0.103	0.00	29	1.000
ADOS 2 (calibrated severity scores)	Social affect	12.2	3.5	11.5	2.8	11.2	3.2	0.27	31	0.790	0.90	29	0.376
	RRB	2.1	1.5	1.9	1.2	3.0	4.1	1.66	33	0.107	−1.02	31	0.316
	Total	14.2	4.0	13.4	3.4	13.2	4.0	0.80	31	0.431	1.04	29	0.307
	Comparison score	7.5	1.8	7.3	1.8	7.1	2.1	0.78	30	0.444	1.09	29	0.283
Social skills measures	TYASSK	17.5	4.3	21.7	3.4	21.4	4.1	−4.71	31	<0.001^**^	−3.67	17	0.002^**^
	SCQ, current	12.0	4.7	11.1	6.0	13.2	8.1	1.10	27	0.279	−0.88	23	0.390
	SSRS by young adults	108.4	26.3	115.7	32.5	109.3	33.3	−2.06	31	0.048^**^	0.85	17	0.410
	SRS-2 by young adults	147.9	24.3	146.5	22.9	147.8	30.8	1.06	30	0.298	−0.73	17	0.477
	SSRS by parents	90.0	22.6	91.8	27.8	99.1	20.7	−0.59	31	0.559	−1.78	24	0.089
	SRS-2 by parents	150.3	19.7	148.6	26.0	136.6	24.5	0.85	31	0.400	5.49	24	<0.001^**^
QSQ by young adults	Hosted get together	1.5	2.3	2.3	2.8	2.8	2.9	−0.84	27	0.408	−1.31	17	0.208
	Hosted date	0.5	1.6	0.2	0.5	0.2	0.8	0.89	24	0.380	0.89	15	0.386
	Invited get together	0.9	1.6	1.3	2.3	2.3	3.7	−0.90	22	0.377	−1.30	13	0.216
	Invited date	0.3	1.0	0.1	0.4	0.3	0.8	0.96	21	0.348	−0.56	12	0.585
QSQ by parents	Hosted get together	1.8	2.4	3.0	3.3	1.7	1.6	−1.74	29	0.093	0.61	20	0.549
	Hosted date	0.7	1.7	0.7	1.4	0.3	0.8	0.38	28	0.710	1.06	17	0.305
	Invited get together	0.9	1.8	1.5	1.4	0.7	1.2	−1.76	23	0.092	0.46	15	0.652
	Invited date	0.3	0.9	0.3	1.0	0.1	0.2	−0.15	22	0.883	1.29	15	0.216
K-VABS	Communication	70.5	16.4	75.0	13.4	80.8	18.3	−2.15	24	0.042^*^	−2.85	23	0.009^**^
	Daily living skills	74.4	20.9	78.3	16.5	83.3	17.2	−0.34	27	0.734	−2.07	24	0.049^*^
	Socialization	65.9	12.7	71.9	18.3	74.8	21.3	−1.70	25	0.101	−1.49	23	0.149
Depressive and anxiety measures	STAI-S	54.0	11.1	53.7	10.6	52.2	8.3	−0.30	30	0.766	0.46	23	0.652
	STAI-T	55.5	10.4	53.3	10.1	49.0	12.6	1.05	30	0.303	2.00	23	0.057
	BAI	31.6	11.5	29.4	8.6	31.2	11.7	1.50	30	0.143	3.14	17	0.006^**^
	BDI	14.5	11.4	12.8	10.2	14.5	9.7	1.80	27	0.084	2.27	13	0.041^*^

* *p <0.05*,

** *p <0.01*.

*ADOS, Autism Diagnostic Observation Schedule; BAI, Beck Anxiety Inventory; BDI, Beck Depression Inventory; FSIQ, Full Scale Intelligence Quotient; K-VABS, Korean version of the Vineland Adaptive Behavior Scale; RRB, Restricted and Repetitive Behaviors; SCQ, Social Communication Questionnaire; SRS-2, Social Responsiveness Scale-2; SSRS, Social Skills Rating System; STAI-S and STAI-T, State and Trait Anxiety Inventory; TYASSK, Test of Young Adult Social Skills Knowledge; QSQ, Quality of Socialization Questionnaire; VCI, Verbal Comprehension Index*.

**Table 4:** *Post-hoc* test with Bonferroni correction, Fisher's LSD, Tukey's studentized range test, Scheffe's contrasts test in the ADOS-2 raw subtotals, and calibrated severity scores.

**Measures**	**Items**	**Pre-test**			**Post-test**		
		**Mean**	**(S.D)**	***post-hoc test***	**mean**	**(S.D)**	***post-hoc test***
ADOS 2 (module 4)	Communication (a)	3.76	(1.28)	b > a > c,d (*p* < 0.05)	3.44	(1.25)	b > a > c,d (*p* < 0.05)
	Social interaction (b)	8.89	(2.02)		8.78	(2.42)	
	Stereotyped behaviors and restricted interests (c)	1.06	(0.23)		1.06	(0.24)	
	Imagination (d)	0.62	(1.01)		0.42	(0.55)	
ADOS 2 (calibrated severity scores)	Social affect (e)	12.56	(3.08)	g > e > h > f (*p* < 0.05)	12.08	(3.63)	g > e > h > f (except Scheffe's test, g,e > h > f) (*p* < 0.05)
	RRB (f)	2.14	(1.49)		1.92	(1.23)	
	Total (g)	14.67	(3.62)		13.91	(3.94)	
	Comparison Score (h)	7.82	(1.64)		7.42	(1.87)	

*ADOS, Autism Diagnostic Observation Schedule; RRB, Restricted and Repetitive Behaviors*.

#### Maintenance of Treatment Effects: 4 Month Follow-Up Assessment: Whole Group Analysis

Social knowledge improvement, as measured by the TYASSK, was found to be maintained after a 4 month follow up (*p* < 0.05). Additionally, the total scores of SRS-2 recorded by parents showed significant improvement 4 months post-treatment (*p* < 0.05). The social interaction domain of the ADOS-2 and the communication and daily living skills of the K-VABS showed statistically significant changes after 4 months of the PEERS^®^-YA-K treatment compared to baseline score (*p*'s <0.05). There were also significant improvements in emotional outcome variables, including the BAI and BDI total scores (*p*'s <0.05). Table 3 shows the treatment maintenance outcomes 4 months after the PEERS^®^-YA-K treatment.

#### Comparison According to Participants' Involvement

The PEERS^®^-YA-K intervention emphasizes completion of weekly homework for the generalization of the skills learned during the treatment sessions. The effectiveness of the treatment according to the homework completion rate was compared to determine how steadfast the participants were in performing the task and if homework completion had an effect on the outcome. The homework completion rate was calculated by the amount of homework the participant completed for each session and was averaged out over all the rates of the sessions. Two to six homework assignments were provided in each session. They were calculated as 100% if they were all completed and 0% if they were not completed at all. The mean completion rate was 50.5% (SD = 17.39) for TG and 46.1% (SD = 18.7) for the DTG; the median was 43.8 and the range of the completion rates was 26–88%. The effect of homework completion on the results was analyzed by dividing the homework completion rate by above and below the median. As a result, higher homework completion was correlated with higher scores on the TYASSK, the socialization domain of the K-VABS, and SSRS. Table 5 shows the treatment effects according to homework completion rate.

**Table 5:** Comparison of treatment effects according to homework completion rate (below/above median).

		**Mean difference**		**Paired** ***t*****-test results for the group with higher completion**		
		**(Post-pre)**		
		**High (*n* = 16)**	**Low (*n* = 17)**	***t***	**df**.	***p*-value**
ADOS 2 (module 4)	Communication	0.0	−0.4	0.00	15	1.000
	Social interaction	−0.7	−0.1	1.65	15	0.119
	Stereotyped behaviors and restricted interests	0.0	−0.1	–	–	–
	Imagination	−0.4	−0.1	1.96	15	0.069
ADOS 2 (calibrated severity scores)	Social affect	−0.4	0.1	0.52	15	0.609
	RRB	−0.3	−0.3	1.07	15	0.300
	Total	−0.6	−0.2	0.72	15	0.482
	Comparison Score	−0.3	−0.1	0.72	15	0.483
Social skills measures	TYASSK	3.6	4.4	−3.02	15	0.009^**^
	SCQ, current	−3.5	0.5	1.67	12	0.120
	SSRS by young adults	10.4	1.1	−2.41	15	0.029^*^
	SRS-2 by young adults	−5.7	−0.7	1.75	15	0.100
	SSRS by parents	4.2	1.1	−0.56	15	0.586
	SRS-2 by parents	−7.3	1.4	1.36	15	0.195
QSQ by young adults	Hosted get together	0.9	0.1	−0.76	13	0.459
	Hosted date	−0.6	0.2	1.34	11	0.206
	Invited get together	0.7	0.0	−0.92	12	0.377
	Invited date	−0.5	0.1	1.20	10	0.258
QSQ by parents	Hosted get together	1.1	1.1	−1.52	14	0.150
	Hosted date	−0.2	−0.1	0.34	13	0.736
	Invited get together	0.8	0.4	−1.70	11	0.117
	Invited date	0.1	0.0	−0.15	10	0.887
K-VABS	Communication	3.8	−3.1	−1.38	12	0.193
	Daily living skills	5.6	−0.2	−1.17	13	0.261
	Socialization	8.3	1.6	−2.21	12	0.047^*^
Depressive and anxiety measures	STAI-S	0.6	0.5	−0.22	15	0.826
	STAI-T	−2.2	−0.9	0.94	15	0.361
	BAI	−7.4	2.6	3.47	15	0.003^**^
	BDI	−3.3	−2.2	1.76	14	0.101

* *p <0.05*,

** *p <0.01*.

*ADOS, Autism Diagnostic Observation Schedule; BAI, Beck Anxiety Inventory; BDI, Beck Depression Inventory; FSIQ, Full Scale Intelligence Quotient; K-VABS, Korean version of the Vineland Adaptive Behavior Scale; RRB, Restricted and Repetitive Behaviors; SCQ, Social Communication Questionnaire; SRS-2, Social Responsiveness Scale-2; SSRS, Social Skills Rating System; STAI-S and STAI-T, State and Trait Anxiety Inventory; TYASSK, Test of Young Adult Social Skills Knowledge; QSQ, Quality of Socialization Questionnaire; VCI, Verbal Comprehension Index*.

## Discussion

This study evaluated the effectiveness of the social and emotional variables of the Korean version (PEERS^®^-YA-K) in young adults with ASD. Through an intensive interview with multiple typically developing adults recruited from the community, we observed that the specific contents and curriculums of PEERS^®^-YA are generally acceptable to the ecology of Korean culture, except that several rules and examples in dating etiquette and available social activities should be modified accordingly. The process of the intervention suggest that the PEERS^®^-YA-K is feasible for application for young adults with ASD in Korea. This notion is reflected by the overall treatment completion rate of 83.78%, and the reasons for withdrawal were not directly related to the treatment itself. This finding is similar to a previous study of teenagers in Korea participating in PEERS^®^, and the completion rate is even higher than that of an RCT conducted at UCLA, which was 72.27% (16, 20). Treatment satisfaction surveys administered at post-treatment revealed an overall satisfaction rate of 76.57% for young adults, demonstrating that the treatment was generally satisfactory for their needs in improving social skills and helped with everyday difficulties, such as habits and negative behaviors which may make others uncomfortable. Moreover, treatment fidelity was 95%. This high-fidelity rate may be due to the translated manual being generally simple to use, group leaders being trained on the intervention prior to treatment, and the fact that the lessons were conveyed to participants without major difficulties.

In the analysis of group differences in the TG and DTG, social knowledge was consistently maintained, similar to our previous research with PEERS^®^ for Adolescents and other RCTs performed by original authors (16, 20). However, the outcomes of the current study only showed improvement in social skill knowledge; this is different from the results of the study conducted at UCLA, which showed not only social knowledge but also improvements in functional social skills measured by the QSQ and SRS (16, 17). Although the causes for these reduced effects are unknown, they might be attributed to a relatively lower effectiveness of the intervention just upon completion, compared to the follow-up assessments. Additionally, another explanation for weakened results compared to previous studies might be due to the characteristics of the adult population in the Korean community. First, stigma about autism in Korea may result in young adults with ASD feeling excluded from activities that would normally provide opportunities for socialization (40–42). Stigma affects those with ASD from childhood to adulthood, along with their parents and family members. This stigma may cause individuals with ASD to be rejected in social situations. In turn, the repeated experience of rejection may lead those with ASD to be passive in social situations (41–44). Furthermore, parents may also limit their children's social activity to prevent them from being rejected (45). Second, there are arguably insufficient social activities for young adults in Korea. This may be attributed to prioritizing studying over recreational activities, over-committed work and school schedules, and a lack of finances and/or resources to engage in leisure activities (46). Finally, as a common limitation to social skills training interventions, appropriate measures that evaluate the social skills associated with ASD are not available. In some studies, role-play tasks as a direct observational assessment were used as an outcome measure in adults with ASD (47–49). However, a systematic contextualized assessment of social skills has yet to be developed, and there are no measures in the standardized Korean version for the contextualized assessment of social skills. In addition, although many studies have used SSRS, most studies did not show treatment effects. Although various measures were used in this study, it is believed that there are insufficient to accurately assess the effectiveness of the treatment.

Results of the comparison between pre-treatment and post-treatment in social knowledge and overall social skills as rated by young adults themselves, as well as social responsiveness and adaptive communication skills as rated by parents, showed significant improvements. It might be noteworthy that the changes observed immediately following treatment were more improved 4 months after treatment completion. In addition to the variables which showed significant improvement after treatment, new improvements were observed in social interaction domains directly observed by the ADOS-2, 4 months after completing the program. This finding suggests that the participants were not only doing well socially after the treatment, they were doing better. New improvements in social skills might be attributed to the effect of practice and repetition of the skills in the community, along with the support of parents as social coaches. The notion of including parents and other caregivers as social coaches in social skills interventions like PEERS^®^ is that the intervention never ends so long as the coach is there to provide support through continuous practice and reinforcement (13). In particular, there were improvements in the communication and daily living skills domain of K-VABS and no significant improvement in the socialization domain, which may be the effect of the joint efforts accomplished by young adults and their parents who attended the PEERS^®^-YA-K together. In other words, the parent-young adult relationship may have improved. This is expected to have a beneficial effect on the parents' continuing intervention as a social coach. The investigators of a 12-week RCT of group training of social skills for children and adolescents with ASD in Sweden also emphasized the importance of strategies for maintaining intensive practice and motivation (50). Analysis of treatment effects according to the homework completion rate revealed a wider range of effects in the higher homework completion rate group. In essence, those who practiced more through the completion of homework assignments, ultimately saw greater improvements in social skills knowledge, social skills measured by SSRS and socialization domain of the K-VABS and anxiety. If this tendency is extended to real-life practice following the completion of a social skills curriculum, more exposure and repetition of the skills might provide more benefit to the participants who continue to apply the knowledge. The social knowledge evaluated by the TYASSK showed significant improvement as the RCT performed for original version of PEERS^®^-YA, but there were no significant results for the frequency of social engagement measured by QSQ, which showed significant results in a previous study of PEERS^®^-YA performed by the original authors.

An additional benefit of PEERS^®^-YA-K was in reducing anxiety and depressive symptoms, as measured by the young adults, 4 months following treatment. Although not the primary treatment goal of PEERS^®^, the reduction of anxiety and depression was also observed in the Korean adaptation of the original version of PEERS^®^ for Adolescents. The association between anxiety and social deficits has been found in numerous studies of the ASD population and anxiety is one of the essential treatment modulators in social skills training, including PEERS^®^ (51–53). Reducing anxiety often leads to a decrease in the avoidance of social activities, which creates more opportunities to practice social skills (54). Young adults with ASD frequently experience social isolation and are more likely to participate less in social activities than young adults with intellectual disabilities (55). Group therapy might be helpful for ASD because teaching social skills in a group environment allows participants to use the skills they have learned within the context of the group (56). One RCT on social skills training and cognitive behavioral therapy for 25 youths showed that adolescents with higher pre-treatment anxiety had greater social impairment and more significant improvements in social skills during treatment and at a 3 month follow up (57). Thus, although there were no significant analyses indicating the association between anxiety and social skills knowledge improvement, the results showed stronger effectiveness of PEERS^®^-YA-K in reducing anxiety (Supplementary Table 3). Therefore, we assumed the following. First, reduction in anxiety may have enabled initiation of social activities and application of newly learned social skills knowledge. Second, repetition and practice of newly learned social skills knowledge in real-life settings (i.e., exposures) may have helped reduce social and general anxiety in everyday life. Further investigations are needed to determine the causality of improvement of social skills as well as the inter-relationship between social skills and anxiety. In summary, the within group analyses showed positive effects of improving social skills knowledge on reducing depression and anxiety symptoms. However, it was revealed that improved social skills knowledge did not lead to improvement in functional social skills in the current study. Therefore, future research is needed on potential individual variables that contribute to the improvement of functional social skills after knowledge acquisition.

Despite the positive findings, this study had a few limitations. First, with the exception of the ADOS-2, most of the research results depended on scales reported by the participants. Although the ADOS-2 was not recommended to be used as the main outcome measure, it was chosen in the absence of other alternatives. Second, there were no ratings from an independent third party and we used un-blinded outcomes. This was mostly due to the fact that the majority of young adults in this study were so socially isolated that an independent third-party observer could not be identified. In addition, to our knowledge, there are no standardized observational measures to assess social skills in the real-world setting that targets our clinical population of interest. Third, variables such as work, job-seeking, and studying could not be controlled, although they may have influenced study findings. Fourth, a portion of the participants were on medication, but we did not control its effect. Fifth, due to the small sample size in the study, the statistical power was also low as various variables were controlled, and multivariate analyses were performed. Finally, we used a waitlist control study design that does not have a control group. This type of design has innate limitations that appear inconsistent with the effectiveness of studies. Future research might take these variables into account and examine their influence on predicting treatment outcome.

## Data Availability Statement

The original contributions presented in the study are included in the article/Supplementary Material, further inquiries can be directed to the corresponding author.

## Ethics Statement

The studies involving human participants were reviewed and approved by the study was approved by IRB of Seoul National University Bundang Hospital (IRB no. B-1611/371-303) and the study was registered at ClinicalTrials.gov (identifier: NCT03310775). The patients/participants provided their written informed consent to participate in this study.

## Author Contributions

EL, HY, and J-HK had designed the study. MO, J-HK, KL, JK, SL, BL, SC, and GB administrated the project. MO, GB, and N-HY participated in data acquisition and data analysis. MO made draft the manuscript and figures. HY provided the outlines for the presentation of the manuscript, supervised the study process, and edited the final manuscript. All authors contributed to the article and approved the submitted version.

## Funding

This work has been supported by Seoul National University Bundang Hospital (14-2016-023) and Original Technology Research Program for Brain Science of the NRF funded by the Korean government, MSIT (NRF-2017M3C7A1027467).

## Conflict of Interest

HY receives royalties from Hakjisa for sales of the Korean versions of Autism Diagnostic Observation Scale and Social Communication Questionnaire and from Sigma Press for sales of the Korean version of PEERS^®^ treatment manual for adolescents and PEERS^®^-YA-K manual. EL receives royalties from Routledge for sales of the PEERS^®^ Treatment Manual and PEERS^®^-YA Treatment Manual. The remaining authors declare that the research was conducted in the absence of any commercial or financial relationships that could be construed as a potential conflict of interest.

## Publisher's Note

All claims expressed in this article are solely those of the authors and do not necessarily represent those of their affiliated organizations, or those of the publisher, the editors and the reviewers. Any product that may be evaluated in this article, or claim that may be made by its manufacturer, is not guaranteed or endorsed by the publisher.
