# Supplementary material for: A Randomized Controlled Trial of the Korean Version of the Program for the Education and Enrichment of Relational Skills for Young Adults (PEERS®-YA-K) With Autism Spectrum Disorder: A Pilot Study
Source: Front Psychiatry. 2021 Oct 6;12:730448. doi: 10.3389/fpsyt.2021.730448 (PMC8526555; doi:10.3389/fpsyt.2021.730448)
Supplement: Supplementary file 1 [file Data_Sheet_1.DOCX]

Supplementary material 1.

PEERS^®^-YA-K adaptation and modification

*Adaptation*

In order to further adapt the intervention and incorporate ecologically valid social skills specific to Korean culture, an intensive interview was performed. For extracting culturally sensitive material and creating interview questions, we performed a consensus meeting with the treatment team and other critical members, such as clinical psychology trainees and medical student volunteers, who provided additional young adults’ perspectives. During the first draft of the translation of the PEERS^®^-YA-K Manual 9-12, we identified the contents that were inappropriate or awkward in the Korean culture and created clarifying questionnaires. Ultimately, 33 questions were developed focusing on the dating etiquette of young adults, along with 14 questions concerning social groups/activities and strategies for handling bullying. The questions regarded items that were considered inappropriate in Korean dating culture, as well as items that were not mentioned in the PEERS^®^-YA American Manual. However, these additional items were important for the development of Korean ASD young adults in order to initiate and maintain successful relationships. In addition, questions on where young adults would like to meet a potential date were developed. Also included were questions about the conversation topics often mentioned among young adults, social groups that can initiate friendships, and activities that can be done while “hanging out” with friends in order to learn about the culture of young adults in Korea. Examples of the question are as follows: "Is there a safe and authorized internet dating website or application in Korea?", "How do young adults express interest in people they like in crowded places, such as meetings and events these days?", "What are some examples of praise that can be awkward?", "What social groups are suitable for young adults in this country to make friends? The general content of the manual was also examined to identify how certain skills might be different in Korea. Methods to modify and implement changes were discussed and then applied through adapted didactic lessons and role play scripts, which were modified to be more culturally sensitive and linguistically appropriate.

A total of 29 typically developing young adults were recruited for the intensive interviews through advertisements within the community. The interviewees consisted of 11 males and 18 females with a mean age of 25 years (range 19-34; *SD* = 3.70; 21 college students and 8 company workers). Interviewees were randomly divided into three groups (Group 1 n=7, Group 2 n=10, Group 3 n=12); two research team members performed each interview and each session took 120-180 minutes to cover all the issues in the questions. To maintain confidentiality and attenuate potential bias from disclosure of their identity regarding private experiences about dating, we assigned a pseudonym for every participant and encouraged interviewees to generalize their experience. The interview was approved by the Institutional Review Board (IRB) of Seoul National University Bundang Hospital (IRB no. B-1605/348-301) and all the interviewees provided written informed consent.

All the interview processes were recorded after proper consent. Also, after the interview, the research team categorized the opinion on each item. We selected the common answers in cases with only one answer, for example, when the young adults made a call or exchanged a text to get the number for a date or after a date or the number of times young adults contacted someone who did not answer a text or call. In addition, in cases with variable answers, we accepted various opinions. For example, cases when the young adults dated and involved in common activities or get-togethers. After this process, on discussion with other experts once again, we determined the consistent opinions of the experts (child psychiatrists, clinical psychologists, and special educators). Consequently, several changes were made to the original PEERS® dating lessons for it to be appropriate as per the Korean culture.

*Modifications*

1. The most common way to find romantic partners in Korea is a blind date, through introductions from mutual friends, known as “*sogae-ting*” (“*sogae*” means “introduction” and “*-ting*” indicates “meeting someone”) Thus, culturally specific etiquette related to blind dating was added to the material in Session 9 (Lesson: *Letting someone know you like them*).
2. While online dating apps and websites can be a common way to find a romantic partner in some Western cultures, those electronic methods are mainly used for finding sexual partners in Korea and might be risky because of total anonymity. Therefore, we removed the content about online dating and added specific warnings about online dating apps and websites to the section about safety in Session 10 (Lesson: *Asking someone on a date*).
3. The interviewees agreed that the spacing of calls or texts after receiving a phone number should be quicker in Korea than in the United States. Therefore, “follow up using the two-day rule” was modified to “follow up using the one-day rule” in Session 10. Likewise, “make a follow up call or text two hours after the date, or when the partner arrives home,” in Session 11 (Lesson: *Going on dates*) was modified from the American rule to follow up the “next day.”
4. The unique characteristics of romantic relationships in the U.S. appear to be quite different among Korean young adults. For example, there is no concept of “casual dating” in Korea. With the advent of online dating, the concept of dating multiple people at one time has become the norm. This is not the case in Korea where online dating is atypical. If someone dates multiple people at the same time in Korea, many people think that they are a “player,” despite being forthcoming about it. Thus, the concept of dating multiple people was removed from Session 12 (Lesson: *Dating Do’s and Don’ts*).
5. Within young adult Korean culture, there is a slang term known as, "*some-tada*” (“*some*” meaning “something is happening to them” and “tada” meaning “in the state of”). This term refers to a relationship where you are not someone’s obvious boyfriend or girlfriend, but you are interested in each other and getting closer to being a couple. We incorporated this slang into the rule, "don't assume that you are a couple or in relationship with someone” when you are in the stage of “*some-tada.*”
6. Other relatively minor, but culturally different content and examples, were also modified.
   1. With regard to dating location, we removed the option about someone’s house, as many young people are living with their parents or other family members and it is uncommon to date at home unless they are in a serious relationship.
   2. We added “video room” as an example of a risky place as it is commonly associated with unwanted sexual pressure.
   3. We added several examples of inappropriate dress, considering common errors that socially awkward young adults make in dating.
   4. We added examples of common, but ambiguous and inappropriate compliments in the context of dating, such as “looks young, looks like a celebrity, looks like they have lost weight.” Additionally, we added appropriate compliments, such as mentioning “something they are wearing,” which might include accessories, hairstyles, or nail polish. The latter compliments would be considered socially acceptable in Korea.
7. We also translated words referring to romantic partners with caution, because Korean society has a tendency to regard heterosexual relationships as the default. We translated “your date” into “your boyfriend/girlfriend,” because there is no gender-neutral word referring to someone whom you date, including “romantic partner,” “lover,” or “someone you are dating.”

In addition to adaptations to dating etiquette, other examples of social groups/activities were also modified in Session 3. We also modified the didactic lessons and role play scripts into more culturally sensitive and linguistically appropriate ways. For example, in Session 11 of the original American version of PEERS^®^ for Young Adults, the role play and steps for “Beginning the date” are based on a date at someone’s house. However, as Koreans do not date at someone’s house at first, the content was changed to be at a restaurant or café. The new rules included: (1) Do not be late, (2) Stand up and greet your date when you meet, (3) Do not scan from head to foot, (4) Initiate conversation in conventional ways, and (5) Mention the date plan. The cultural differences in other sessions overlapped with the Korean version of the PEERS^®^ for Teens with ASD. Please refer to this paper for more information (Yoo et al. 2014).

Table S1. Modified contents in Korean version of PEERS^®^-YA

| Session | Topic | Content | Changes to the Korean version of the PEERS^®^-YA |
| --- | --- | --- | --- |
| 8 | Get-togethers | Common Activity | - We re-categorized and added/deleted some social activities as appropriate to the culture of Korean young adults. For example, we added learning activities such as study TOEIC together, preparing certification and studying for job-hunting, indoor sports games such as baseball and golf games, playing computer games at PC parlor, and entertaining at singing room. Warhammer, Lacrosse, Bocce ball, Renaissance fairs were deleted. |
| 9~12 | Dating | Romantic partner | - Korean society has a tendency to regard heterosexual relationships as the default. We translated “your date” into “your boyfriend/girlfriend” |
| 9 | Dating etiquette | Dating sources | - Common way to find romantic partner: blind date through introduction from mutual friends (“sogae-ting”). - Dating website, bar, and club are deleted. |
|  | Letting someone know you like them | Talk to mutual friends | - I like someone 🡪 I am interested in someone. |
|  |  | Give compliments | - Inappropriate compliments: Looks young, looks like celebrity, looks like losing weight. - Appropriate compliments: “something they are wearing”, such as accessories, hairstyles, or nail polish, as reflecting compliments about outfits. - Physical compliments should be from the neck up and hands. |
| 10 | Asking someone on a date | What to do | - Double date, where two couples who are close friends with each other go on a date together. |
|  |  | Where to meet | - Deleted online date: Online dating apps/sites are mainly used for finding sexual partners and it is very easy to disguise their identity . - Deleted someone’s house: It is not common to date at home in Korea as many young adults live with their parents. - Inappropriate dating places (e.g., DVD room, video room: A place for presenting DVDs in closed individual rooms and it can be a vulnerable place for unwanted sexual contact). |
|  |  | Assess their interest | - Say they are busy, but seem disappointed 🡪 Ask for other date. |
|  |  | Follow up using the two-day rule | - Follow up using the one-day rule: Call or text one day after getting someone’s number or asking someone out. |
| 11 | Going on dates | Follow up using the two-day rule | - Follow up using the one-day rule: Young adults in the focus group had a consensus that one day is more appropriate for follow up in our culture. |
|  |  | Dress appropriately | - Inappropriate clothing: Slippers, gold necklaces, too many accessories, uniform, too big or too small clothes, inappropriate clothes for age and weather. |
|  |  | Staying safe on the date | - Drive yourself to and from the date 🡪 If you take your date’s car, then meet or ask them to drop you off at a public place or train and bus station. |
|  |  | Beginning of the date | - As Koreans do not date at someone’s house at first, we changed it to be at a restaurant or café and make new rules, as follows: 1) Do not be late for the date; 2) Stand up and greet your date when you meet; 3) Do not scan from head to foot; 4) Initiate conversation in conventional ways; 5) Mention the date plan. |
|  |  | Do not ignore your date | - Do not send Kakao talk messages (kind of SMS) during date. |
|  |  | Be prepared to pay: Use the two offer rule | - Use the “next time” rule 🡪 Alternating payment for meals in dating or friendly meetings is natural in Korean culture and it is a conventional to say, "I will pay next time", "As you paid for the movie, I will pay for the popcorn", or "As you paid for the meal, I will pay for the dessert (restaurant and dessert places separate in Korea)" |
|  |  | Ending the date | - Do not do or permission for any physical contact or do not do it if you are not sure: In terms of physical contact, Korean young people tend to be more conservative. |
|  |  | Make a follow up call or text the next day | - Make a follow up call or text after two hours. |
| 12 | Dating do’s and do not’s | Do not assume you are a couple | - Ask definite questions that ensure you are a couple (e.g., “Do you want to go on a date?”). |
|  |  | Do not be a player | - There is no concept of “casual dating” in Korea. If someone dates multiple people at the same time, many people think that they are a player, despite being forthcoming about it. Thus, the concept of date with multiple people was deleted. - “some-tada”(“some” meaning “something is happening to them” and “-tada” meaning “in the state of”): You are not someone’s obvious partner, but you are interested in each other and getting closer to being a couple. Thus, we specified "don't assume that you are a couple or in relationship with someone” when you are in the stage of “some-tada”. |
| Whole session | Prospective Taking Questions |  | - Changed the answer of the perspective taking questions for Korean culture. |
