# Supplementary material for: A Randomized Controlled Trial of the Korean Version of the Program for the Education and Enrichment of Relational Skills for Young Adults (PEERS®-YA-K) With Autism Spectrum Disorder: A Pilot Study
Source: Front Psychiatry. 2021 Oct 6;12:730448. doi: 10.3389/fpsyt.2021.730448 (PMC8526555; doi:10.3389/fpsyt.2021.730448)
Supplement: Supplementary file 2 [file Table_1.DOCX]

Supplementary material 2

Table S2. A lesson summary of PEERS^®^-YA-K

| Week | Didactic Lesson | Summary |
| --- | --- | --- |
| 1 | Trading information and starting conversations | 1. Explain about the purpose and instruction of PEERS®-YA-K  2. Rules for trading information  3. Steps for starting conversation |
| 2 | Trading information and maintaining conversations | 1. Common conversational topics  2. Trading information and maintaining conversation |
| 3 | Finding a source of friends | 1. Social groups and finding social activities  2. Finding common interest with people  3. Assessing peer acceptance of rejection |
| 4 | Electronic communication | 1. Steps for exchanging contact information  2. Steps for starting and ending phone calls and leaving voicemail  3. General rules for using electronic communication |
| 5 | Appropriate use of humor | 1. Rules about appropriate use of humor  2. Assessing humor feedback signs |
| 6 | Entering group conversations | 1. Steps for entering group conversations |
| 7 | Exiting conversation | 1. Reasons for not being accepted in conversations  2. Steps for exiting conversations |
| 8 | Get-togethers | 1. Planning get-togethers  2. Common activities for get-togethers  3. Steps for beginning and ending get-togethers |
| 9 | Dating etiquette: Letting someone know you like them | 1. Dating sources  2. Letting someone know you like them |
| 10 | Dating etiquette: Asking someone on a date | 1. Asking someone on a date  2. Steps for accepting rejection and turning someone down |
| 11 | Dating etiquette: Going on dates | 1. Planning and preparing for the date  2. Steps for beginning and ending the date  3. Rules for during and after the date |
| 12 | Dating etiquette: Dating do’s and don’ts | 1. Dating do’s and don’ts  2. Handling sexual pressure from partners |
| 13 | Handling disagreements | 1. Steps for responding to disagreements  2. Steps for bringing up disagreements |
| 14 | Handling direct bullying | 1. Handling teasing and embarrassing feedback  2. Handling physical bullying |
| 15 | Handling indirect bullying | 1. Handling cyberbullying and being the target of gossip  2. Steps for spreading the rumor about yourself |
| 16 | Moving forward and graduation | 1. Suggestions moving forward |
