# Supplementary material for: A Randomized Controlled Trial of the Korean Version of the Program for the Education and Enrichment of Relational Skills for Young Adults (PEERS®-YA-K) With Autism Spectrum Disorder: A Pilot Study
Source: Front Psychiatry. 2021 Oct 6;12:730448. doi: 10.3389/fpsyt.2021.730448 (PMC8526555; doi:10.3389/fpsyt.2021.730448)
Supplement: Supplementary file 3 [file Table_2.DOCX]

Supplementary material 3

Table S3. The relationship between anxiety and social skills in two models

|  | **Multivariate logistic regression** | | **Univariate logistic regression** | |
| --- | --- | --- | --- | --- |
|  | **OR** | **95% CI** | **OR** | **95% CI** |
| TYASSK | 0.699 | (0.092-5.341) | 0.787 | (0.183-3.387) |
| SCQ, current | 0.888 | (0.122-6.480) | 3.333 | (0.831-13.372) |
| SSRS by young adults | 4.230 | (0.396-45.137) | 5.555 | (0.864-35.702) |
| SRS-2 by young adults | 0.099 | (0.009-1.051) | 0.436 | (0.097-1.958) |
| SSRS by parents | 1.872 | (0.261-13.453) | 1.484 | (0.370-5.957) |
| SRS-2 by parents | 1.207 | (0.178-8.209) | 1.956 | (0.425-8.999) |

SCQ: Social Communication Questionnaire; SRS-2: Social Responsiveness Scale-2; SSRS: Social Skills Rating System; TYASSK: Test of Young Adult Social Skills Knowledge

* The first model is the result of a multivariate analysis that corrected for age, FSIQ, mediation, and compliance. The second model is the result of a single-variable analysis that included each independent variable.
